# Enhanced Photon Emission Efficiency Using Surface Plasmon Effect of Pt Nanoparticles in Ultra-Violet Emitter

**DOI:** 10.3390/mi10080528

**Published:** 2019-08-09

**Authors:** Hee-Jung Choi, Sohyeon Kim, Eun-Kyung Chu, Beom-Rae Noh, Won-Seok Lee, Soon-Hwan Kwon, Semi Oh, Kyoung-Kook Kim

**Affiliations:** 1 Department of Advanced Convergence Technology, and Research Institute of Advanced Convergence Technology, Korea Polytechnic University, 237 Sangidaehak-ro, Siheung-si 15073, Korea; 2 Department of Electrical Engineering and Computer Science, University of Michigan, Ann Arbor, MI 48109, USA; 3 Department of Nano-optical engineering, Korea Polytechnic University, 237 Sangidaehak-ro, Siheung-si 15073, Korea

**Keywords:** ultraviolet (UV) emitter, surface plasmon, Pt nanoparticles, hole-pattern, photon emission efficiency

## Abstract

We demonstrate the surface plasmon (SP)-enhanced ultraviolet (UV) emitter using Pt nanoparticles (NPs). The UV emitter is hole-patterned on the *p-*AlGaN layer to consider the penetration depth of Pt NPs. The Pt NPs with sizes under 50 nm are required to realize the plasmonic absorption in UV wavelength. In this study, we confirm the average Pt NP sizes of 10 nm, 20 nm, and 25 nm, respectively, at an annealing temperature of 600 °C. The absorption of annealed Pt NPs is covered with the 365-nm wavelength. The electroluminescence intensity of SP-UV is 70% higher than that of reference UV emitter without hole-patterns and Pt NPs. This improvement can be attributed to the increase of spontaneous emission rate through resonance coupling between the excitons in multiple quantum wells and Pt NPs deposited on the *p*-AlGaN layer.

## 1. Introduction

Ultraviolet (UV) photon emitters are currently in high demand for applications including lighting, lamps, chemical and biological agent detection, sterilization, and many medical uses [1,2,3]. However, the UV emitters have more technical problems enhancing the light efficiency than visible emitters because of the shorter wavelength of the UV emitters, such as the light absorption in *p*-AlGaN layer and transverse magnetic mode emission [4,5,6,7]. Therefore, it is necessary to improve the total light efficiency in UV emitters.

Recently, surface plasmon (SP) have drawn great attention for their ability to enhance the photon emission efficiency (PEE) of the emitter [8,9,10,11,12,13]. SPs are the collective oscillations of free electrons at a metal-dielectric interface [14]. The SP coupled between metal and semiconductor creates an alternative emission channel besides the intrinsic emission through carrier recombination. Therefore, it has been reported that the metal nanostructures could play a significant role in improving the PEE of emitters by SP coupling [15,16].

Among these metallic nanostructures, nanoparticles (NPs) are of great interest and have demonstrated their usefulness in PEE enhancement by controlling the energy transfer between multiple quantum wells (MQWs) and SP [13,15,16]. However, the metal NPs buried inside the UV emitters for approaching the MQWs can even deteriorate the epitaxial quality of the MQWs and, consequently, degrade the electrical characteristics of devices. The suppression of emission might be caused by the small size and low height due to strong resonant absorption and poor photon extraction, as reported in [17,18,19,20]. Therefore, these NPs are usually formed by the rapid thermal annealing (RTA) of metals, and the challenges lie in accurately controlling their shapes and dimensions. Furthermore, the penetration depth, which effectively causes plasmonic resonance, is determined for each metal material [21]. The closer the metal particles are formed in the MQWs region, the more effectively both the internal quantum efficiency and the PEE of the photon emitter can be increased.

In this study, we demonstrate the enhancement of the photon efficiency of the UV emitter using Pt NPs. For high plasmonic resonance, we fabricated periodic hole-patterns on top of the *p*-AlGaN layer of UV emitter. The finite difference time domain (FDTD) simulations were also conducted to compare the experimental results.

## 2. Materials and Methods

**Figure 1:**
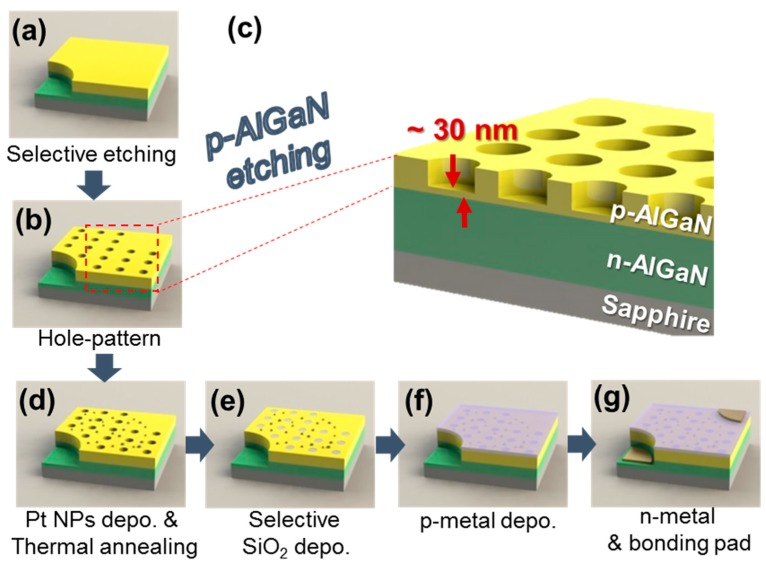
(**a**–**g**) Schematic diagram of surface plasmon-ultraviolet (SP-UV) emitter with the periodic hole-patterns and Pt nanoparticles (NPs).

Figure 1 shows a schematic diagram and the fabrication procedure of SP-UV emitter. The epitaxy layers of UV emitter were grown on a sapphire substrate by metal-organic chemical vapor deposition.

The UV emitter (350 × 350 μm^2^ in chip size) consisted of a 2 nm-thick *p*-GaN:Mg layer, a 0.1 μm-thick *p*-AlGaN:Mg (n_a_ = 5 × 10^17^ cm^−3^) layer, a 20 nm-thick AlGaN electron blocking layer, a 100 nm-thick MQWs with In_0.002_Ga_0.998_N/Al_0.04_Ga_0.96_N active layers, a 2.0 μm-thick *n*-Al_0.08_Ga_0.92_N:Si (n_d_ = 5 × 10^18^ cm^−3^) layer, and a 2.0 μm-thick un-doped GaN layer on the sapphire substrate. 

In order to fabricate the device, the epitaxy layer was selectively etched by an inductively coupled plasma (ICP) etching process until the *n*-AlGaN layer was partially exposed. The *p*-AlGaN layer was hole-patterned through the photolithography and ICP etching process. Then, Pt NPs were formed on the hole patterned *p*-AlGaN layer using the RTA process at 600 °C annealing temperature. After Pt NPs deposition, selective SiO_2_ was deposited using an electron-beam evaporator. The ITO layer was deposited by an electron-beam evaporator on the *p*-AlGaN layer and annealed at 600 °C in O_2_ ambient for 1 min using RTA. The Ti (50 nm)/Al (200 nm) layers were deposited as an *n*-electrode and annealed at 550 °C in N_2_ ambient.

For comparison, a conventional UV (C-UV) emitter (without any pattern and Pt NPs), a periodic hole-patterned UV (HP-UV) emitter, and an SP-UV emitter sample (with HPs and Pt NPs) were simultaneously prepared to compare the light emission property. The penetration depth of Pt NPs is defined by the following formula [21].

(1) $$\mathrm{Penetration}\mathrm{depth}=\frac{\lambda }{2\pi }\sqrt{\frac{\epsilon_{d}-\epsilon_{m}}{\epsilon_{m^{2}}}}$$

The λ is the wavelength of the emitter, $\epsilon_{d}$ is the dielectric constant of dielectric material, and $\epsilon_{m}$ is the dielectric constant of metal. Using this formula, we can calculate that the penetration depth of the Pt layer is about 45 nm at the 365-nm wavelength [22]. Based on this calculation, the *p*-AlGaN layer was periodically hole-patterned with the 5 μm diameter and 90-nm thickness (30 nm-thick *p*-AlGaN layer was remained under the holes).

The Pt layers were deposited on top of the hole-patterned UV emitter by sputtering for 60 s (2 nm-thick), 90 s (3 nm-thick), and 120 s (4 nm-thick) in a vacuum of 0.09 mbar, respectively (thickness data not shown). The deposited Pt layers were annealed at 500 °C, 600 °C, 700 °C, and 800 °C, respectively, to realize the Pt NPs.

Pt NPs were examined by Field Emission scanning electron microscopy (FE-SEM) (Hitachi, S-4100) and atomic force microscopy (AFM) (Park systems, XE-100). The photoluminescence (PL) intensity was measured using the laser excited with DPSS (Diode Pumping Solid State) (λ = 266 nm) and 30 mW power. The PL intensity was detected by Ocean Optics USB 2000. The current–voltage (*I–V*), Light output intensity–injection current (*L–I*), and electroluminescence (EL) measurements of fabricated UV emitters were carried out using a parameter analyzer (Keithley-2420 source meter), and Ocean optics-US 4000 spectrometer with the 0.1–10.0-nm full width and half maximum (FWHM) resolution.

## 3. Results and Discussion

**Figure 2:**
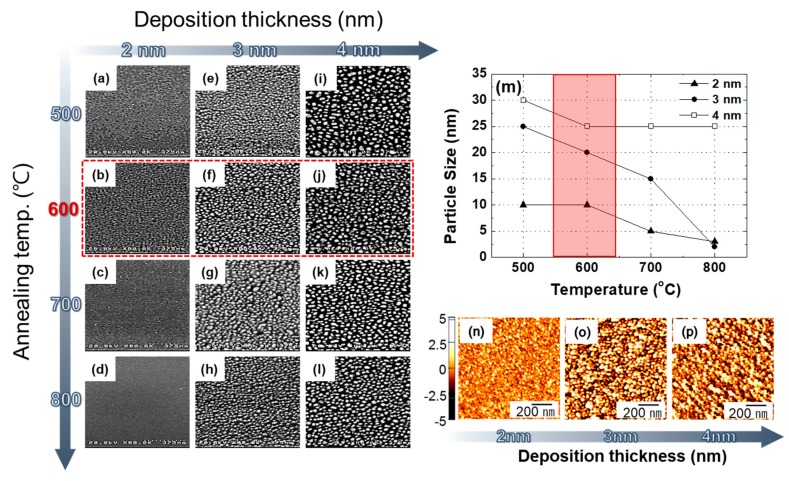
SEM images of Pt NPs with different deposition thicknesses and annealing temperatures. (**a**–**d**) 2 nm, (**e**–**h**) 3 nm, and (**i**–**l**) 4 nm-thick Pt layers with 500–800 °C annealing temperatures; (**m**) Average diameters of Pt NPs with annealing temperatures; (**n**–**p**) AFM images of annealed 2–4 nm of Pt layers at 600 °C annealing temperature.

The SEM images of Pt NPs with different deposition thicknesses and annealing temperatures are shown in Figure 2. Figure 2a–l show the Pt NPs with 2 nm (sputtered for 60 s), 3 nm (sputtered for 90 s), and 4 nm-thick Pt layers (sputtered for 120 s) at different annealing temperatures, respectively. As the Pt thicknesses are increased, the Pt NPs sizes are also increased. The average NPs sizes as annealing temperatures are shown in Figure 2m. As shown in this graph, the Pt NPs sizes are decreased with increasing annealing temperatures. Several research groups reported the change of absorption peak positions as Pt NPs sizes [18,19]. On the basis of some experimental results, we fabricated Pt NPs under 50-nm diameters for application to UV emitter. At annealing temperature of 600 °C, we can confirm the average NPs sizes are 10 nm (2 nm deposition), 20 nm (3 nm deposition), and 25 nm (4 nm deposition), respectively. Thus, we measured the electrical and optical properties of UV emitter with Pt NPs annealed at 600 °C.

Figure 2n–p show the AFM images of Pt layers annealed at 600 °C. The average root mean square (RMS) of Pt NPs are shown 0.883, 1.285, and 1.353, respectively. As the thickness of the Pt layers becomes thicker, the RMS of the Pt NPs are also increased. These results show a similar tendency with the surface images of Pt NPs as shown in Figure 2.

**Figure 3:**
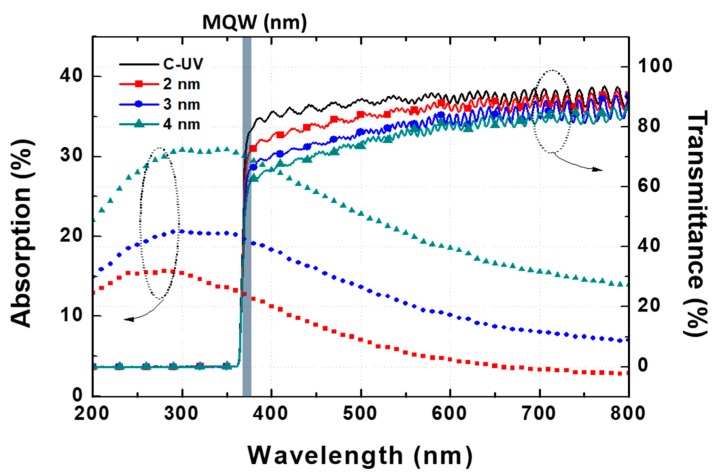
Absorption and transmittance of the Pt NPs annealed at 600 °C.

The absorption and transmittance of C-UV and SP-UV with the Pt layers annealed at 600 °C are measured. The reflectance of all Pt NPs was only near 0.2%. Therefore, the reflectance is not considered in this experiment. Figure 3 shows that the thickness of the Pt layer are increased, the absorption is also increased. The absorption peaks of Pt NPs are located near the 300-nm wavelength region with the broadband spectrum. Therefore, the absorption tail covers the 365-nm wavelength region. The absorption peak is red-shifted with increasing diameter of NPs due to the aspect-ratio dependence of the localized surface plasmon resonance [18,19,23,24]. In contrast to other materials, such as gold and silver, this linearity holds even for small particle sizes and makes Pt a highly interesting material to apply in the optoelectronic devices [14,15,16]. The decrease of the absorption intensity of Pt NPs is connected with the fact that the coverage is smaller for smaller particles, which implies a smaller number of electrons undergoing interband transitions, thus yielding a lower absorption intensity [18,19].

**Figure 4:**
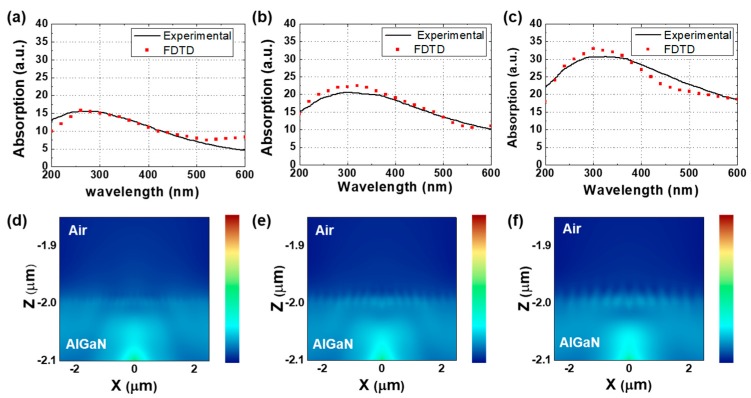
Finite-difference time-domain (FDTD) simulations according to the diameters of Pt NPs in UV emitters. Calculated and experimented absorption and field propagations according to the diameters of Pt NPs (**a**,**d**) 10 nm, (**b**,**e**) 20 nm, and (**c**,**f**) 25 nm, respectively.

Figure 4 shows finite-difference time-domain (FDTD) simulations according to the diameters of Pt NPs on UV emitters. The Pt NPs are located on the *p*-AlGaN layer of the UV emitter, and the dipole source is set into the AlGaN layer with the 30-nm distance from air region. The refractive index of Pt NPs follows Palik model [25,26,27,28]. The size and distance of the Pt NPs have 10 nm, 20 nm, and 25 nm, respectively, similar to the SEM images of Figure 2. The absorption of Pt NPs on UV emitter was estimated using the transmittance and reflectance from the simulation results of Figure 4a–c. As shown in these simulation results, the absorption is also increased and red-shifted as the diameters of Pt NPs increased. The absorbed peaks simulated by the FDTD are almost the same as the experimental data. The field propagation images are also shown in Figure 4d–f. The UV emitter with Pt NPs (10-nm diameter) has higher light propagation than other UV emitters. It is expected that the light absorption in 10 nm is lower than the other particle sizes and has a higher light scattering center [18,19,20,23,24].

**Figure 5:**
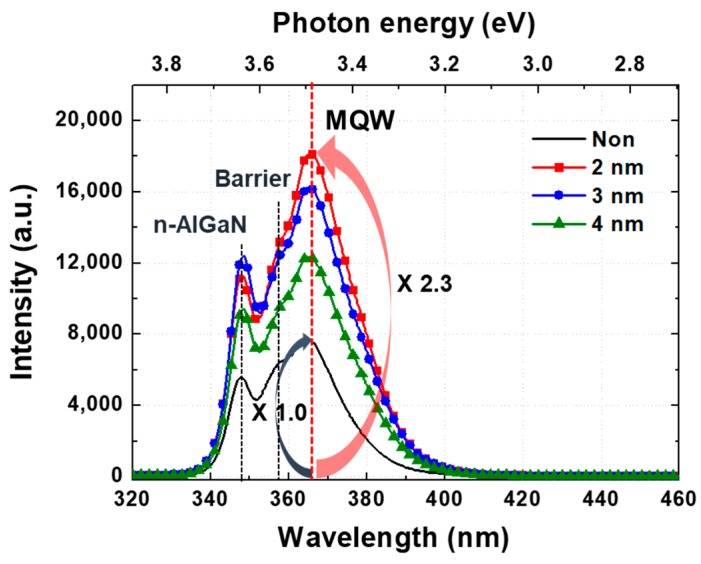
PL results of UV emitters with different sizes Pt NPs.

Before measurement the electrical and optical properties of emitters, we confirmed the PL properties of UV emitters in Figure 5. The 366-nm peak is strongly shown in all samples, that is, the photon emitted from the active layer in the emitters. The peak measured at 358 nm is inferred to the photon emitted from the barrier located between the QW active layers. The peak observed at 348 nm is considered to the photon emitted from the *n*-AlGaN layer. The UV emitter without Pt NPs shows the lower PL intensity than those with Pt NPs. The PL intensity of the UV emitters with the 2 nm, 3 nm, and 4 nm-thick Pt layers were 2.3, 2, and 1.6 times higher than without Pt NPs. This enhancement can be attributed to the strong coupling by SP between the MQWs and the Pt NPs. The PL intensity is decreased as Pt thickness is increased because the intensity of the laser reaching to the MQWs is reduced as the Pt NPs sizes are increased. The photon emitted by the laser is also reflected and absorbed by the Pt NPs on the surface of UV emitter [29,30,31].

**Figure 6:**
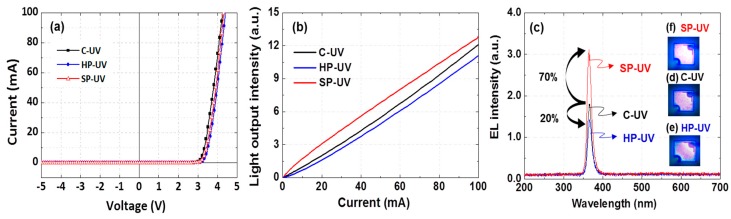
(**a**) *I–V*, (**b**) *L–I*, and (**c**) EL intensity of C-UV, HP-UV, and SP-UV emitters. (**d**–**f**) Emission images of UV emitters at an injection current of 20 mA.

In order to investigate the electrical and optical properties of C-, HP, and SP-UV emitters, *I–V*, *L–I*, and EL characteristics were measured. The *I–V* curves in Figure 6a show that the voltages of C-UV, HP-UV, and SP-UV are 3.5, 3.7, and 3.6 V at an injection current of 20 mA, respectively. This shows that HP-UV and SP-UV samples with HP have a slightly higher series resistance than C-UV sample because of the surface defect during formation of hole-patterns. In addition, the decrease of electrical property is partly attributed to the resistance increase from the reduction of *p*-AlGaN volume [32]. Figure 6b shows the *L–I* characteristics of the UV emitters. The SP-UV shows the highest light output intensity in every injection current than other UV emitters. The EL intensities for each sample with an injection current of 20 mA are shown in Figure 6c. The EL intensity of HP-UV is 20% lower than that of C-UV and SP-UV is 70% higher than that of C-UV. This is because the HP-UV emitter is reduced of the electrical properties and caused the current crowing due to etching damage by fabrication of hole-patterns. If we realize the more stable current spreading in HP-UV chip, the light intensity of the HP-UV emitter can enhance light emission compared to C-UV. The large light enhancement of SP-UV is due to the SP effect, which occurred between Pt NPs and the photons emitted from UV emitter. Figure 6d–f also shows the emission images of UV emitters with the same injection current. Although the emission image of HP-UV is slightly darker than that of C-UV, the SP-UV chip has a brighter emission image compared with C-UV chip. However, we can confirm that this damage can be overcome by SP effect using Pt nanoparticles, although the SP-UV has also electrical damage during the etching process.

## 4. Conclusions

In conclusion, we demonstrate the enhanced PEE of the 365-nm wavelength UV emitter using the Pt NPs. In order to effectively use the SP effect to the emitter, the sizes of NPs are controlled by deposition thickness of Pt layers and the annealing process. At 600 °C annealing temperature, we realized the 10 nm, 20 nm, and 25 nm sizes of Pt NPs. In addition, to consider penetration depth of Pt NPs, we fabricated periodic hole-patterns on top of the *p*-AlGaN layer. Through the absorption data, we can find that all NPs have an absorption intensity near the 300-nm wavelength and have the broadband absorption spectrum covered with the 365-nm wavelength. We confirmed that the PL intensity of UV emitter with the 10-nm sizes of Pt NPs was 2.6 times higher than that without Pt NPs. The small size of Pt NPs (10 nm) has lower light absorption and higher light scattering center compared with the other sizes of Pt NPs (20 nm and 25 nm). Through the *I–V* results, we can confirm the HP- and the SP-UV emitters have a slightly higher series resistance than C-UV emitter because of the etching damage of *p*-AlGaN layer for periodic hole-patterns. However, the EL intensity shows that the SP-UV emitter has a higher intensity of 70% than that of C-UV emitter. This large enhancement is because of the strong coupling by SP between the MQWs and the Pt NPs.
